# Integrative analyses of prognosis, tumor immunity, and ceRNA network of the ferroptosis-associated gene FANCD2 in hepatocellular carcinoma

**DOI:** 10.3389/fgene.2022.955225

**Published:** 2022-09-29

**Authors:** Zhihao Yang, Yaoshu Song, Ya Li, Yiming Mao, Guobo Du, Bangxian Tan, Hongpan Zhang

**Affiliations:** ^1^ Department of Oncology, Affiliated Hospital of North Sichuan Medical College, Nanchong, China; ^2^ Tianjin Key Laboratory of Medical Epigenetics, Key Laboratory of Breast Cancer Prevention and Therapy (Ministry of Education), Department of Biochemistry and Molecular Biology, Tianjin Medical University, Tianjin, China; ^3^ North Sichuan Medical College, Nanchong, China; ^4^ Department of Pathology and Medical Research Center, Beijing Chaoyang Hospital, Capital Medical University, Beijing, China; ^5^ Suzhou Kowloon Hospital, Shanghai Jiao Tong University School of Medicine, Suzhou, China

**Keywords:** HCC, ferroptosis, FANCD2, prognosis, ceRNA, immunotherapy

## Abstract

Extensive evidence has revealed that ferroptosis plays a vital role in HCC development and progression. Fanconi anemia complementation group D2 (FANCD2) has been reported to serve as a ferroptosis-associated gene and has a close relationship with tumorigenesis and drug resistance. However, the impact of the FANCD2-related immune response and its mechanisms in HCC remains incompletely understood. In the current research, we evaluated the prognostic significance and immune-associated mechanism of FANCD2 based on multiple bioinformatics methods and databases. The results demonstrated that FANCD2 was commonly upregulated in 15/33 tumors, and only the high expression of FANCD2 in HCC was closely correlated with worse clinical outcomes by OS and DFS analyses. Moreover, ncRNAs, including two major types, miRNAs and lncRNAs, were closely involved in mediating FANCD2 upregulation in HCC and were established in a ceRNA network by performing various *in silico* analyses. The DUXAP8-miR-29c-FANCD2 and LINC00511-miR-29c-FANCD2 axes were identified as the most likely ncRNA-associated upstream regulatory axis of FANCD2 in HCC. Finally, FANCD2 expression was confirmed to be positively related to HCC immune cell infiltration, immune checkpoints, and IPS analysis, and GSEA results also revealed that this ferroptosis-associated gene was primarily involved in cancer-associated pathways in HCC. In conclusion, our investigations indicate that ncRNA-related modulatory overexpression of FANCD2 might act as a promising prognostic and immunotherapeutic target against HCC.

## Introduction

Hepatocellular carcinoma (HCC) remains the sixth and third leading cause of cancer-related morbidity and death globally among men and women (Sung et al., 2021; Siegel et al., 2022). HCC accounts for 75–85% of primary liver cancer cases, with an increasing incidence in China and the United States (Islami et al., 2017; Tapper and Parikh, 2018; Zhou et al., 2019). Despite advances in traditional treatment methods, including surgical resection, orthotopic liver transplantation, and locoregional therapies, the survival of patients with HCC is still particularly poor (Forner et al., 2018; Llovet et al., 2021). Currently, four forms of immune checkpoint inhibitors have proven to be promising and effective immunotherapy strategies for HCC (Killock, 2017; Vogel and Saborowski, 2022). However, only a subset of patients can benefit from immunotherapy, with a generally efficient response rate of no more than 20% (Cheng et al., 2020; Sangro et al., 2020). Our main aim is to identify immune-modulatory biomarkers for diagnosis, prognosis, and treatment of HCC.

Fanconi anemia complementation group D2 (FANCD2) belongs to one of the DNA repair markers, which is a class of Fanconi anemia (FA) proteins that regulate DNA damage responses and maintain genomic integrity (Liu et al., 2010; Semlow and Walter, 2021). Recent studies have demonstrated that FANCD2 in HCC cells maintains resistance to DNA-damaging tumor suppression by increasing DNA repair activity, FANCD2 deletion restricts chemoresistance of HCC cells *in vitro* (Palagyi et al., 2010), and elevated FANCD2 is closely linked to adverse prognosis in HCC (Komatsu et al., 2017), glioma cells (Chen et al., 2021), breast cancer (Fagerholm et al., 2013), colorectal cancer (Ozawa et al., 2010), and non-small-cell lung cancer (Ferrer et al., 2005). FANCD2 has been reported to elicit an active role in the negative regulation of ferroptosis (Song et al., 2016), a recently new form of programmed cell death characterized by iron-dependent peroxide lipid accumulation, leading to many forms of tumors that are sensitive to ferroptosis induction treatment (Dixon et al., 2012). However, the immune-regulating effect on this ferroptosis-associated gene in HCC onset and progression remains unexplored.

In our current study, we deciphered the role of ferroptosis-associated gene FANCD2 expression and prognosis in pancancers anchored by several databases. Subsequently, ncRNAs, including two major types, miRNAs and lncRNAs, were found to be closely involved in mediating FANCD2 in HCC and were analyzed through ceRNA. Finally, a high level of FANCD2 in HCC was also positively associated with immune cell infiltration, immune checkpoints, and IPS analysis, suggesting that ncRNA modulatory upregulation of FANCD2 is an immune-associated biomarker with an adverse influence on HCC patient clinical survival.

## Methods

### Data collection and expression

The expression data of FANCD2 and corresponding clinical information in 33 types of tumors were processed from UCSC Xena (Navarro Gonzalez et al., 2021) (https://xena.ucsc.edu/) using R software (Version 4.0.3; https://www.R-project.org) and Strawberry Perl (5.3.0.1). The expression of FANCD2 in HCC was also verified by the Integrative Molecular Database of Hepatocellular Carcinoma (HCCDB), which contains 15 public HCC expression datasets and provides candidate-targeted gene expression prognosis and coexpression analysis (http://lifeome.net/database/hccdb/search.html) (Lian et al., 2018). Furthermore, the Cancer Cell Line Encyclopedia (CCLE) database was used to determine the diverse expression of FANCD2 in HCC cell lines (https://portals.broadinstitute.org/ccle/about) (Barretina et al., 2019).

### starBase online database and lncRNA subcellular locations

The starBase database (http://starbase.sysu.edu.cn/) (Li et al., 2014) was applied to verify FANCD2 expression and prognostic status in many tumors. Then, this website, including PITA, RNA22, miRmap, microT, miRanda, PicTar, and TargetScan, was adopted to predict the upstream miRNA of target genes (FANCD2) and the expression and survival status of screened miRNAs in HCC. Subsequently, to ascertain candidate lncRNAs *via* a competing endogenous RNA (ceRNA) mechanism, the upstream lncRNAs of the miR-29c-3p-FANCD2 axis were predicted, and the web was conducted to examine lncRNA expression and its correlation with FANCD2 together with miR-29c-3p. Finally, Cytoscape software (v3.6.0) was used to visualize the FANCD2-regulated ceRNA network.

Arched by binomial distribution approaches, the iLoc-lncRNA database (Su et al., 2018) and lncLocator database (Lin et al., 2021) were applied to accurately predict the subcellular locations of lncRNAs, encompassing four locations of lncRNAs in a cell (nucleus, cytoplasm, ribosome, and exosome).

### Overall survival and disease-free survival analyses

Kaplan–Meier (KM) survival curves, including overall survival (OS) and disease-free survival (DFS) analyses, combined with a log-rank test mapped differences of FANCD2 for all types of TCGA tumors employing the GEPIA tool (http://gepia.cancer-pku.cn) (Tang et al., 2019), which was validated by the HCCDB (Lian et al., 2018). *p* < 0.05 was treated as the significance threshold for all statistical analyses.

### Analysis of tumor immunity aimed at Fanconi anemia complementation group D2

The association between FANCD2 expression and infiltrating immune cells, such as B cells, CD4^+^ T cells, CD8^+^ T cells, neutrophils, macrophages, and dendritic cells, was analyzed across the HCC patients captured from the TIMER2 web server (https://cistrome.shinyapps.io/timer/) (Li et al., 2020), which was incorporated into 10,897 samples from TCGA.

Immunotherapy has proven to be an effective and promising strategy against tumors, especially involving the use of CTLA-4, PD-1, and PD-L1 antibodies to treat several types of tumors (Galluzzi et al., 2020). Therefore, we used the TIMER2 database to explore the interplay between FANCD2 and these immune checkpoints. The immunophenoscore (IPS), which can be obtained from the TCIA website (https://tcia.at/home), has been verified to predict the patients’ response to immune checkpoint inhibitors (ICIs). A higher IPS has a better outcome with ICI treatment (Charoentong et al., 2017).

### Gene set enrichment analysis

Gene set enrichment analysis (GSEA) (Subramanian et al., 2005), including Gene Ontology (GO) and Kyoto Encyclopedia of Genes and Genomes (KEGG) functional enrichment analyses, was used to determine the potential mechanisms and functions of FANCD2 in HCC, with the following parameters: nPerm = 1,000, minGSSize = 10, maxGSSize = 1,000, and nominal *p-*value < 0.05.

## Results

### Overview of the workflow

**FIGURE 1:**
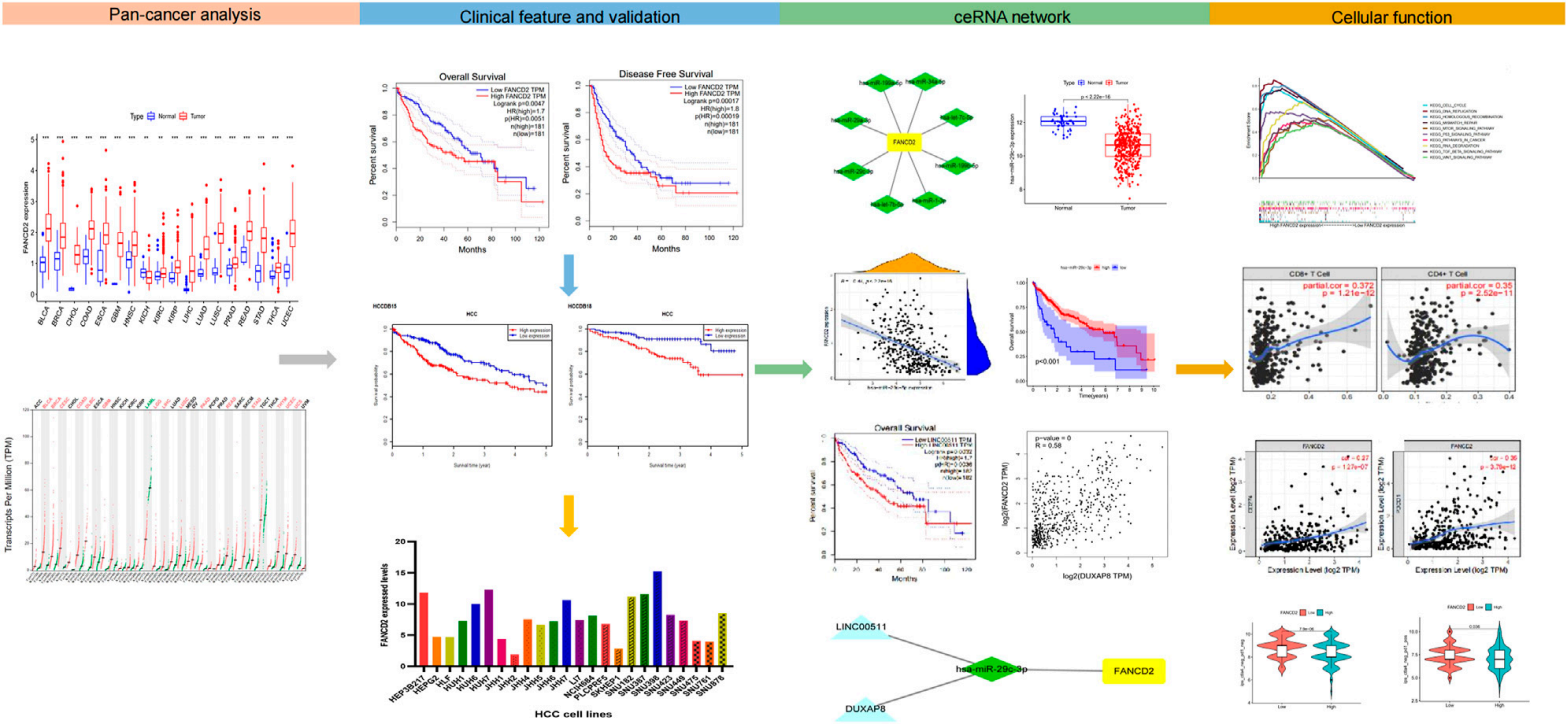
Research workflow of construction and analysis of the FANCD2 ceRNA network.

Our goal was to detect the potential function of FANCD2 in various TCGA cancer types and to investigate the correlation of immunotherapy between FANCD2 and HCC (Figure 1). To this end, we collected various public data sources and conducted a series of bioinformatics and functional genomic analyses.

### Fanconi anemia complementation group D2 mRNA expression across cancers

**FIGURE 2:**
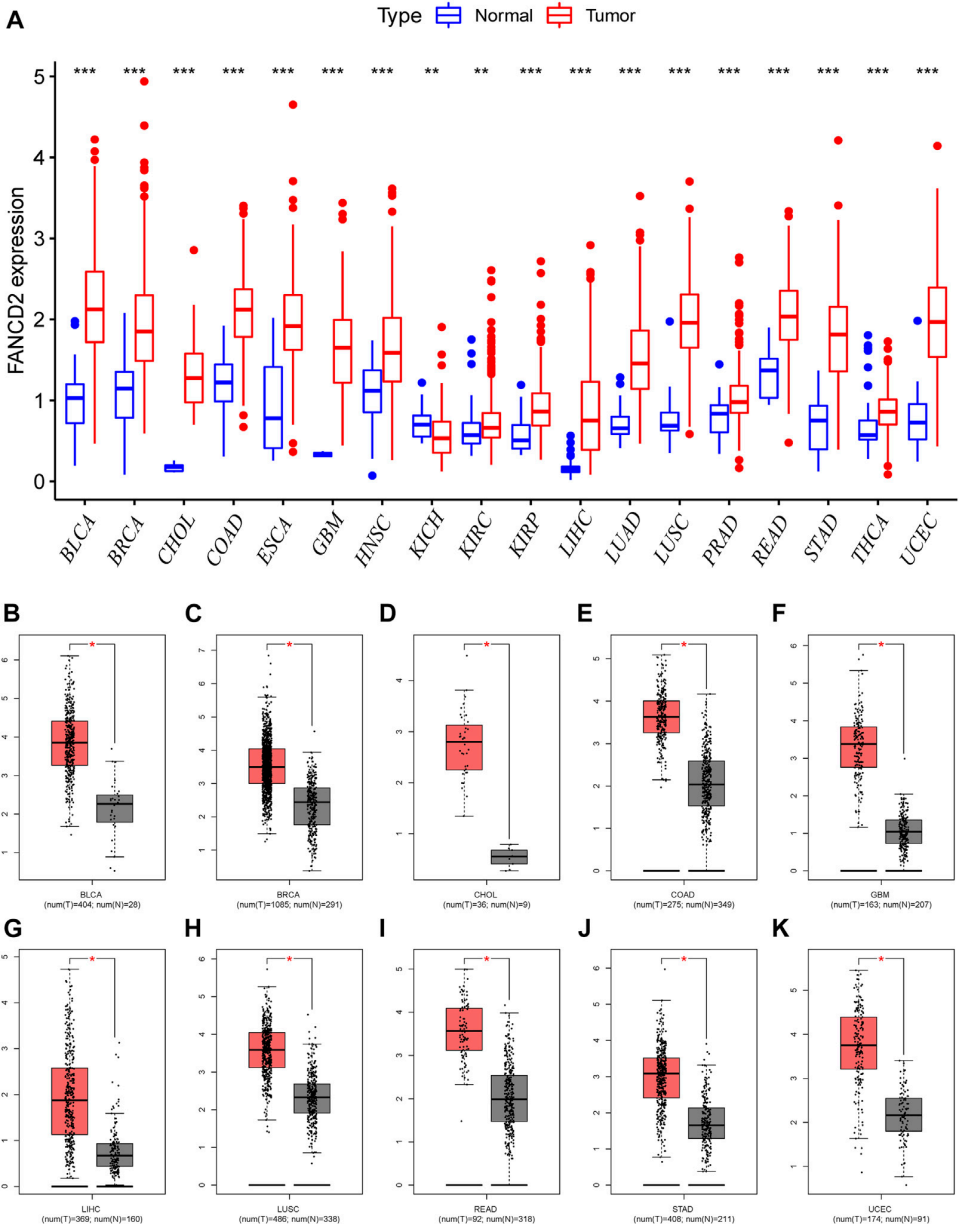
Pancancer expression profiling analysis of FANCD2. **(A)** Interactive body map of FANCD2 mRNA expression constructed by GEPIA. **(B–K)** Expression of FANCD2 was analyzed between TCGA cancer tissues and GTEx normal tissues (*p < 0.05, **p < 0.01, ***p < 0.001).

Aberrantly expressed analysis from TCGA data revealed that FANCD2 was differentially expressed in many human tumor tissues, and that FANCD2 mRNA overexpression was found in BLCA, BRCA, CHOL, COAD, ESCA, GBM, HNSC, KIRC, KIRP, HCC, LUSC, PRAD, THCA, and UCEC, whereas FANCD2 loss was found in KICH (Figure 2A). We further evaluated whether FANCD2 expression is high in most human cancers compared with matched normal samples utilizing the starBase database. This result indicated that FANCD2 mRNA expression in BLCA, BRCA, CHOL, COAD, GBM, HCC, LUSC, READ, STAD, and UCEC was also high (Figures 2B–K), suggesting that FANCD2 acts as an oncogene in multiple tumors, which was also verified by the HCCDB (Supplementary Figure S1A and Table 1).

**TABLE 1:** Differential expression analysis of FANCD2 from clinical cohorts impinged on the HCCDB.

Dataset	*p*-value	Type	Nums	Mean	STD	IQR
HCCDB1	1.81E-22	HCC	100	6.201	0.7713	1.172
		Adjacent	97	5.196	0.3941	0.5852
HCCDB15	5.24E-29	HCC	351	7.058	1.367	2.175
		Adjacent	49	4.691	0.8484	0.95
HCCDB16	1.09E-08	HCC	60	5.841	0.7623	0.9007
		Adjacent	60	5.17	0.2636	0.2963
HCCDB18	2.45E-44	HCC	212	1.537	0.6642	0.9425
		Adjacent	177	0.6843	0.333	0.35

### Prognostic implication of Fanconi anemia complementation group D2 in screened cancers

**FIGURE 3:**
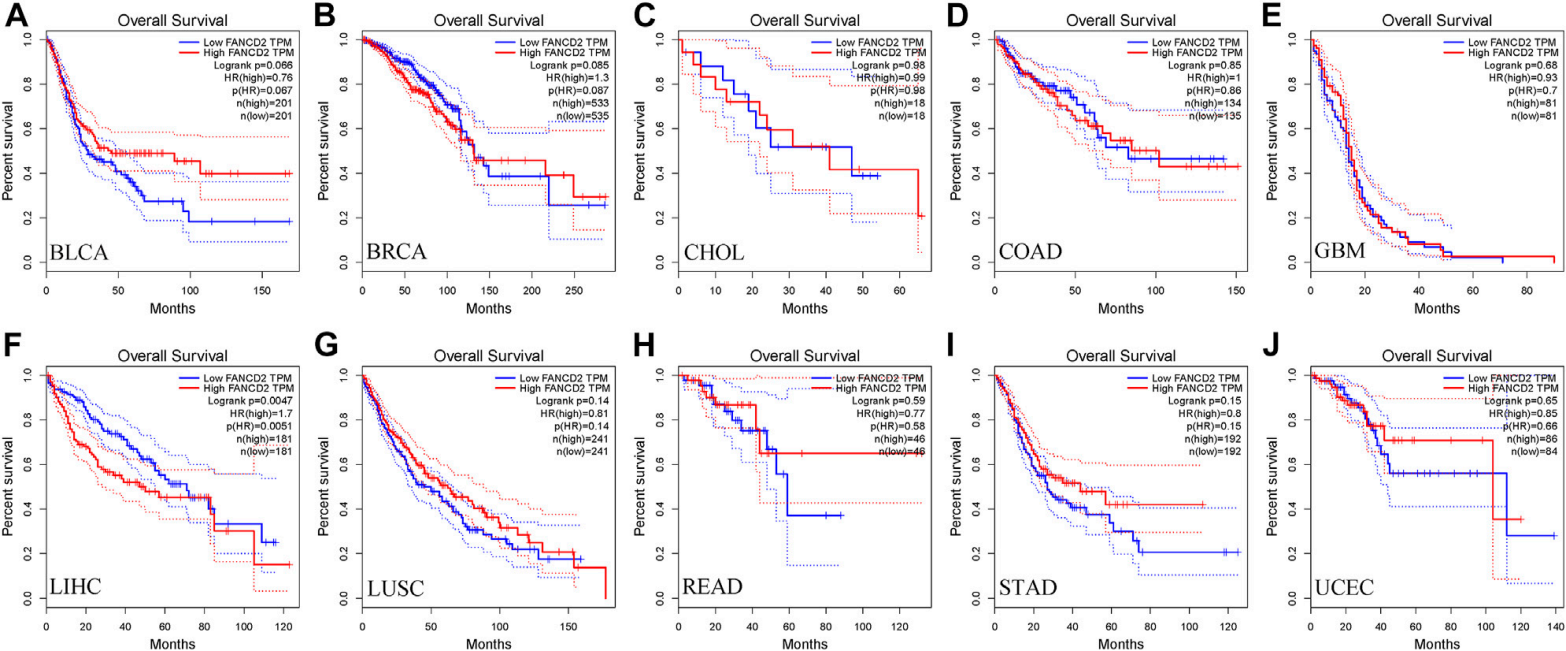
Correlation of FANCD2 expression with patient OS. **(A–J)** K-M method showed that high expression of FANCD2A was closely correlated with shorter lifetimes in BLCA, BRCA, CHOL, COAD, GBM, LIHC, LUSC, READ, STAD, and UCEC.

**FIGURE 4:**
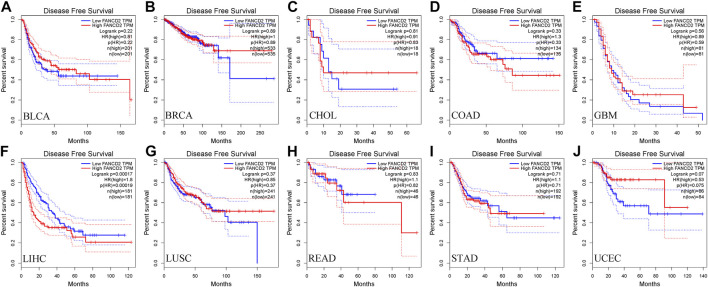
Correlation of FANCD2 expression with patient DFS. **(A–J)** K-M method showed that increased expression of FANCD2A was closely related to disease-free survival lifetimes in BLCA, BRCA, CHOL, COAD, GBM, LIHC, LUSC, READ, STAD, and UCEC.

Next, we used the GEPIA database within the RNA sequencing data from TCGA and GTEx data to ascertain the prognostic implication of FANCD2, including OS and DFS, in the aforementioned 10 cancers (BLCA, BRCA, CHOL, COAD, GBM, HCC, LUSC, READ, STAD, and UCEC). The results indicated that only the high level of FANCD2 showed worse OS in HCC (*p* = 0.0047, HR = 1.7, Figure 3), which was consistent with DFS in HCC (*p* = 0.00017, HR = 1.8) (Figure 4). Intriguingly, we also used the CCLE database and found that FANCD2 in most HCC cell lines had at least partly high levels (Supplementary Figure S1B). The finding that high FANCD2 activity in HCC was associated with unfavorable OS (*p* = 0.00767 and 0.00325, Supplementary Figures S1C,D) indicates that overexpressed FANCD2 might be a crucial prognostic factor in HCC.

### Prediction and analysis of upstream miRNAs of Fanconi anemia complementation group D2

**FIGURE 5:**
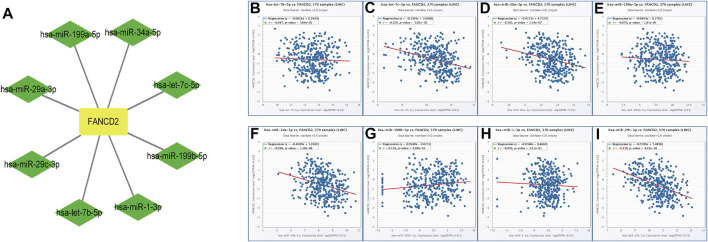
Identification of the miRNA-FANCD2 regulatory network in HCC. **(A)** FANCD2-associated miRNA network was visualized using Cytoscape. **(B–I)** Expression correlation between predicted miRNAs and FANCD2 in HCC analyzed by the starBase database.

**FIGURE 6:**
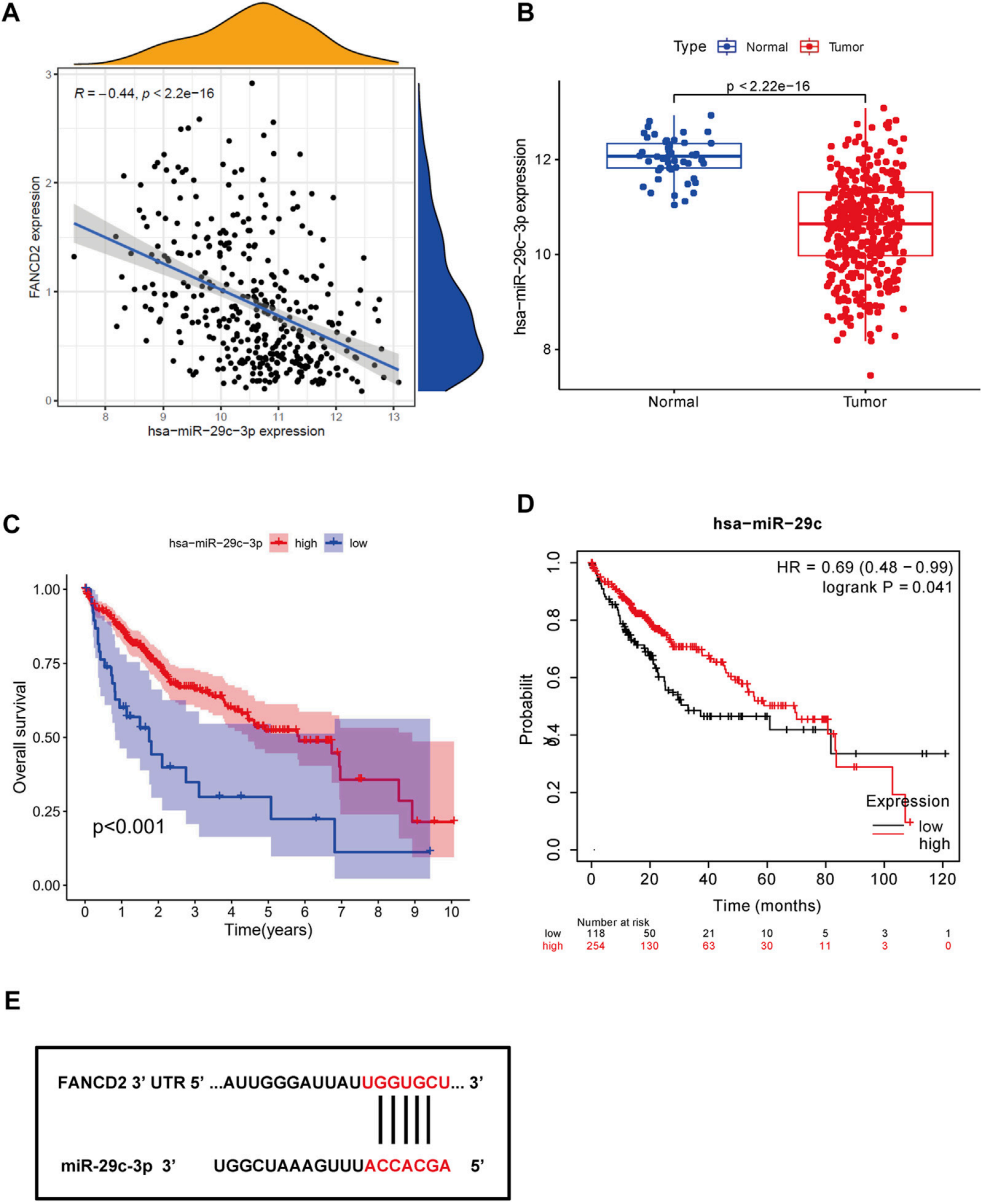
Identification of miR-29c-3p as the most potential regulatory miRNA of FANCD2 in HCC. **(A)** Association between FANCD2 and miR-29c-3p expression is shown in the dot plot. **(B)** Expression distribution of miR-29c-3p in HCC. **(C,D)** Kaplan‒Meier survival analysis of miR-29c-3p. **(F)** Base pairing of miR-29c-3p of the 3′ UTR in FANCD2-binding sites.

Non-coding RNAs, including miRNAs and lncRNAs, that account for nearly 98% of transcriptomes have long been thought of as pivotal players in the biological regulation of cancer cells, such as ceRNA and gene expression (Beermann et al., 2016). Therefore, our first aim was to identify possible miRNAs involved in modulating FANCD2. Notably, miRNAs directly bind to the 3′-untranslated region (UTR) of the corresponding target mRNA, leading to their repression or posttranscriptional gene silencing (Goodall and Wickramasinghe, 2021). These findings revealed that eight screened miRNAs directly interact with FANCD2 upstream (Figure 5A) and are negatively associated with hsa-let-7c-5p, hsa-miR-29a-3p, hsa-miR-34a-5p, and hsa-miR-29c-3p in HCC *via* correlation analysis (*p* < 0.05, Figures 5B–I). Among these miRNAs, hsa-miR-29c-3p was markedly distinct from FANCD2 expression (Figure 6A). Intriguingly, low hsa-miR-29c-3p in HCC revealed unfavorable patient OS through the K-M plotter database, and it was significantly low in the aforementioned tumors (Figures 6B–D). The 3’ UTR arising from FANCD2 that targeted miR-29c-3p was also successfully predicted by the starBase database (Figure 5E). These outcomes suggested that hsa-miR-29c-3p is the most likely regulatory miRNA of FANCD2 in HCC.

### Prediction and analysis of upstream lncRNAs of hsa-miR-29c-3p

**FIGURE 7:**
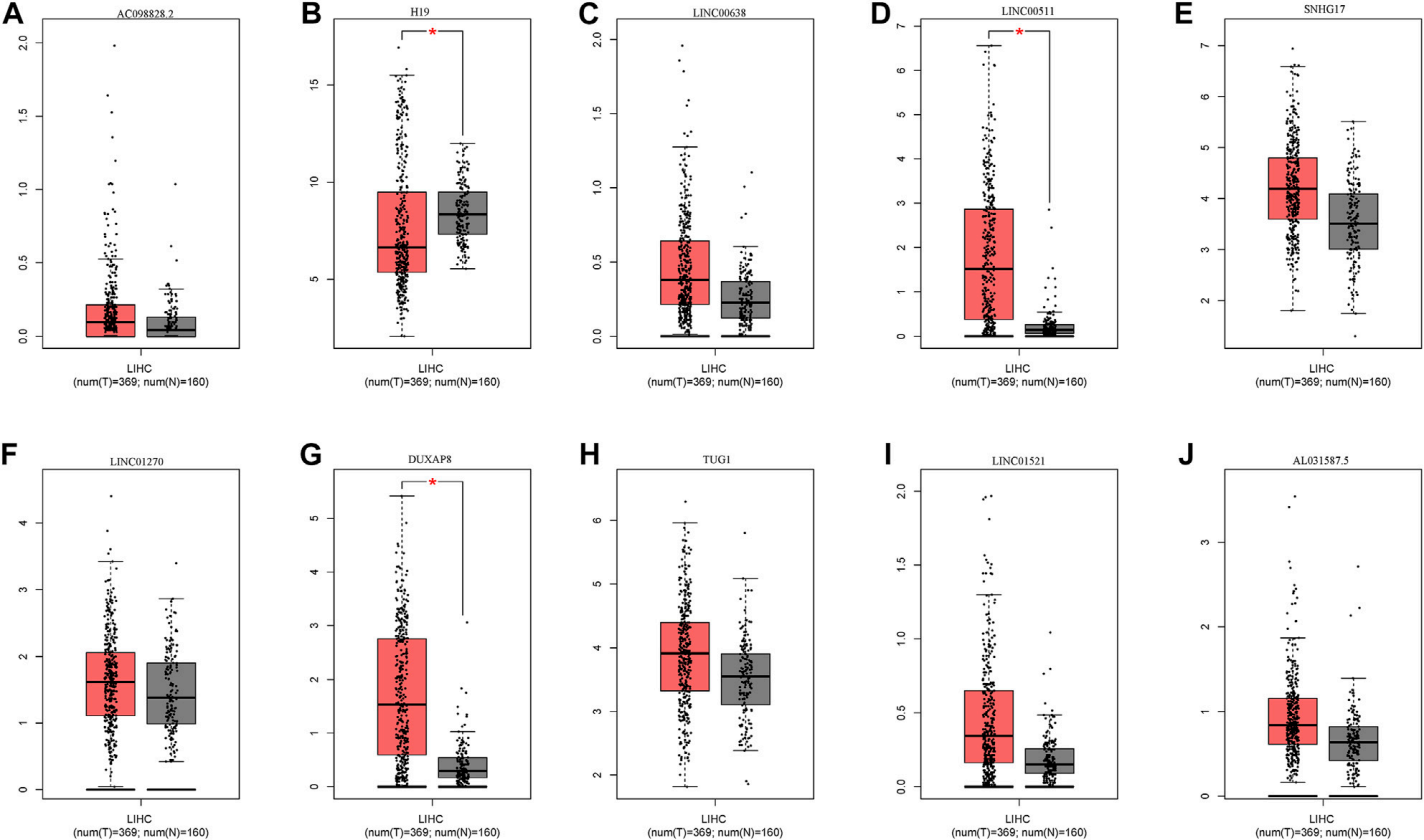
Expression of upstream lncRNAs predicted by miR-29c-3p. **(A)** AC098828.2, **(B)** H19, **(C)** LINC0068, **(D)** LINC0051, **(E)** SNHG17, **(F)** LINC01270, **(G)** DUXAP8, **(H)** TUGI, **(I)** LINC01521, **(J)** AL031587.5.

**FIGURE 8:**
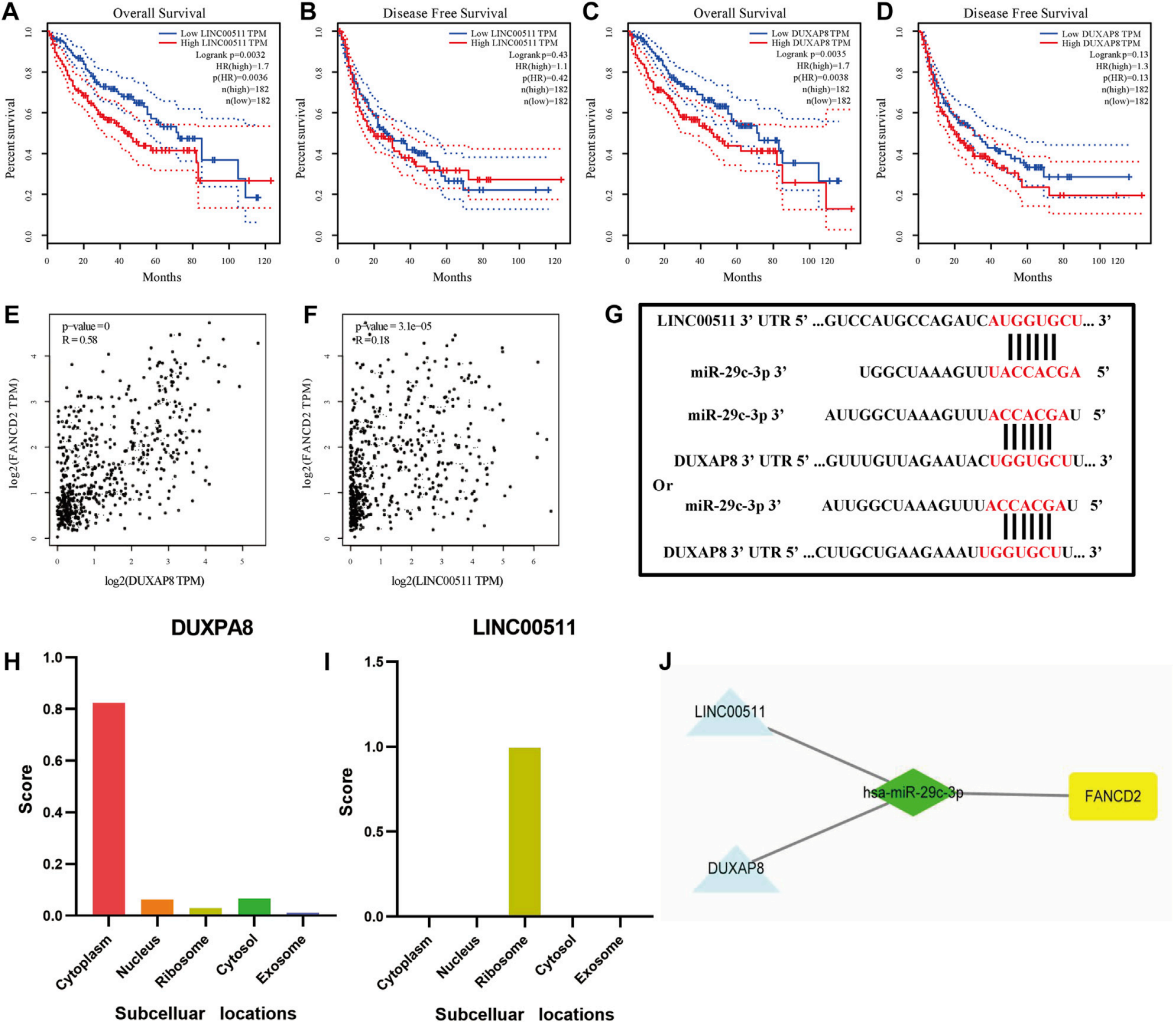
DUXAP8 and LINC00511 are the most likely upstream lncRNAs of the hsa-miR-29c-3p-FANCD2 axis in HCC. **(A–D)** Kaplan‒Meier survival analysis of DUXAP8 and LINC00511 through OS and DFS analyses. **(E,F)** Comparison of DUXAP8 and LINC00511 and FANCD2 expression in HCC. **(G)** Base pairing of DUXAP8 and LINC00511 of the 3′ UTR in miR-29c-5-binding sites. **(H)** Prediction of DUXAP8 and LINC00511 subcellular localization using lncLocator. **(I)** FANCD2-associated ceRNA network was visualized using Cytoscape.

In addition to screened miRNAs, we predicted hsa-miR-29c-3p-modulatory lncRNAs in HCC using the starBase database and observed eight possible lncRNAs (Figures 7A–J). Anchored by the ceRNA hypothesis, lncRNAs have been viewed as sponges of miRNAs to restrict the reduction in the role of target mRNAs (Salmena et al., 2011). Accordingly, DUXAP8 and LINC00511 in HCC were elevated compared with those in normal samples, and their high levels were reciprocally relevant to better patient OS among all eight lncRNAs (Figures 8A–D). More importantly, DUXAP8 and LINC00511 were strongly associated with FANCD2 expression but were antithetical to hsa-miR-29c-3p, according to the starBase database (Figures 8E,F).

To determine whether these two lncRNAs were involved in regulating FANCD2-based ceRNAs, we used the lncLocator database and the iLoc-lncRNA database to analyze their subcellular localization. As seen in Figures 8H,I and Supplementary Figures 2A,B, DUXAP8 was mainly distributed in the cytoplasm, while LINC00511 was mostly located in the ribosome, indicating that the former in HCC are more likely to participate in the FANCD2-specific ceRNA network. The target sequence of the 3’ UTR of DUXAP8 and LINC00511 paired with miR-29c-3p was predicted *via* the starBase online database (Figure 8G). To this end, both lncRNAs most likely are upstream lncRNAs of the hsa-miR-29c-3p-FANCD2 axis in HCC, especially DUXAP8 (Figure 8J).

### Fanconi anemia complementation group D2 induces immune infiltration in the hepatocellular carcinoma tumor microenvironment

**FIGURE 9:**
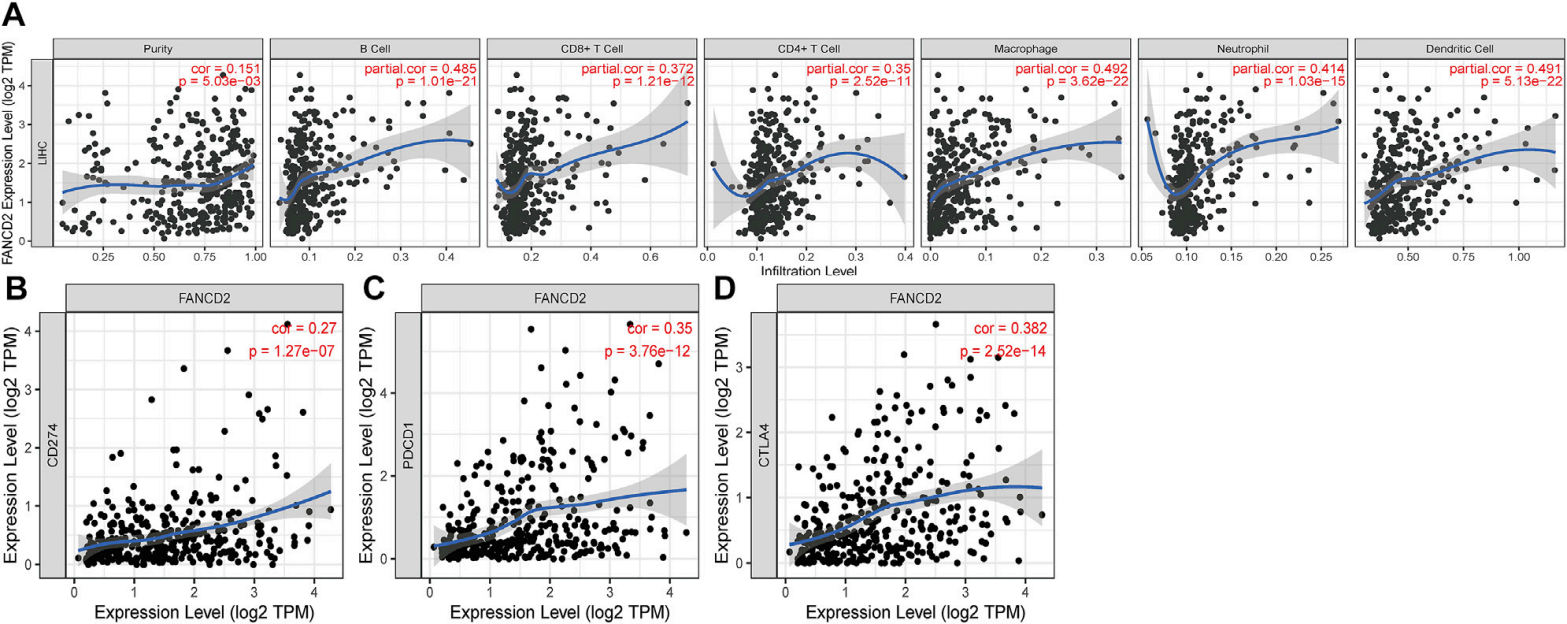
Correlation of FANCD2 expression with immune infiltration level **(A)** and immune checkpoints **(B–D)** in HCC.

It has been well documented that infiltrating immune cells exert a crucial role in the tumor microenvironment (TME), which can regulate the initiation and development of cancers (Su et al., 2018; Cózar et al., 2021; Jhunjhunwala et al., 2021). When we examined the tumor immunity of FANCD2, we found that the FANCD2 expression level in HCC showed a significant interaction with immune infiltration (Figure 9A). As shown in Figure 9A, the FANCD2 expression level in HCC had an enormously positive correlation with neutrophils, purity, macrophages, CD4^+^ T cells, CD8^+^ T cells, dendritic cells, and B cells, indicating that FANCD2 could be of great importance in the regulation of the immune infiltration function of HCC.

### Interaction between Fanconi anemia complementation group D2 and immune checkpoints and IPS analysis

Immune checkpoints, including PD-1/PD-L1 and CTLA-4, have gained attention in cancer-associated therapy *via* immune surveillance and escape (Dall'Olio et al., 2022; Doroshow et al., 2021). We ascertained the role of the immune response of FANCD2 in HCC with PD-1, PD-L1, or CTLA-4 using TIMER data and found that FANCD2 expression was strongly statistically associated with PD-1, PD-L1, and CTLA-4 in HCC (Figures 9B–D), demonstrating that FANCD2 might mediate HCC carcinogenesis of immune escape.

**FIGURE 10:**
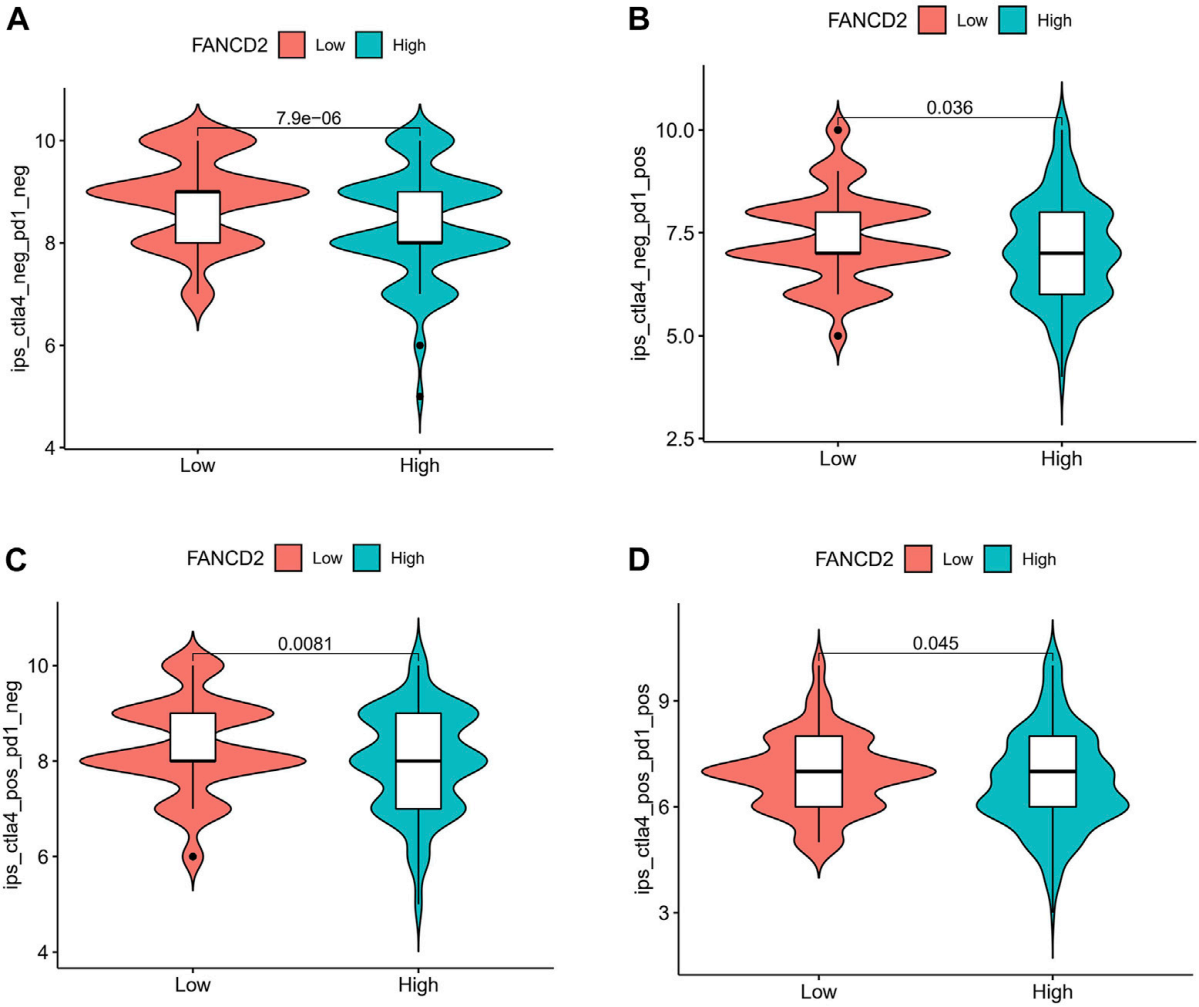
Correlation of FANCD2 expression with the immunophenoscore (IPS) in HCC. **(A)** ips_ctla4_neg_pd1_neg, **(B)** ips_ctla4_neg_pd1_pos, **(C)** ips_ctla4_pos_pd1_neg, **(D)** ips_ctla4_pos_pd1_pos.

The IPS values (ips_ctla4_neg_pd1_pos, ips_ctla4_pos_pd1_neg, ips_ctla4_pos_pd1_neg, and ips_ctla4_pos_pd1_pos) have been validated in predicting cancer patients’ responses to anti-CTLA-4 treatment. Here, we noted a marked rise in scores in the low-FANCD2 group (Figures 10A–D; *p* < 0.05), implying that the low-FANCD2 group is more suitable for anti-CTLA-4 and anti-PD-1/PD-L1 immunotherapy.

### Enrichment analysis of Fanconi anemia complementation group D2

**FIGURE 11:**
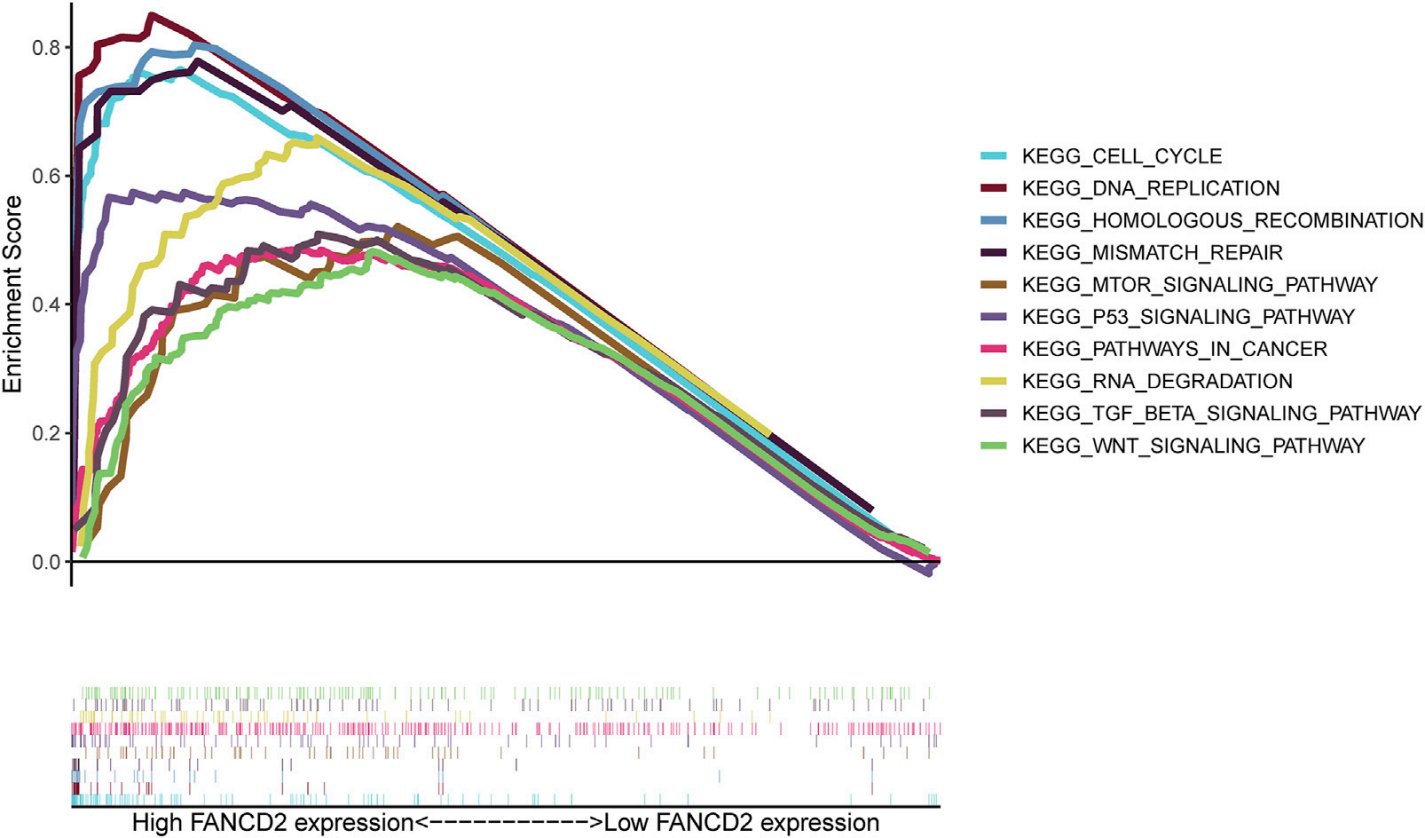
GSEA functional enrichment of FANCD2 in HCC.

To predict the functions or pathways that FANCD2 in HCC may modulate, GSEA was carried out. The results showed that activated FANCD2 was positively enriched in the cell cycle, p53 signaling pathway, mTOR signaling pathway, DNA replication, TGF-beta signaling pathway, Wnt signaling pathway, and RNA degradation (*p* < 0.05; Figure 11), revealing the tumor-associated and even tumor-promoting roles of FANCD2 and shedding light on its potential mechanisms in HCC.

## Discussion

HCC is a malignant tumor that threatens human health (Llovet et al., 2021). Despite advancements in immunotherapy against HCC, only a minority of patients are eventually able to prolong their survival time (Cheng et al., 2020; Faivre et al., 2020; Pinter et al., 2021). The identification of better immune-associated prognostic biomarkers for timely detection, early diagnosis, and treatment of HCC is urgently needed. Accumulating evidence has shown that FANCD2 can significantly resist ferroptosis-related concentrations of iron and lipid ROS in various diseases (Song et al., 2016; Li et al., 2021), acting as a ferroptosis-related regulator. Ferroptosis can also regulate immune activity within the HCC microenvironment (Zhao et al., 2020). Nevertheless, little progress has been achieved in studying the roles and mechanisms of this ferroptosis-related gene against HCC.

Our current study investigated whether the ferroptosis-related gene FANCD2 in the pancancer analysis was more activated than in counterpart tissues, as validated in the starBase database, other databases, and many published articles. Survival analysis further revealed that only FANCD2 at a high expression level had shorter OS and DFS survival in HCC, indicating that this gene could serve as a potential and prognostic target in this deadly cancer.

It has been well documented that lncRNAs can act as ceRNA networks by relieving the suppressive effects of targeted genes through miRNAs to facilitate their expression (Salmena et al., 2011; Tay et al., 2014). Our study found that there were eight possible miRNAs that contained binding sites for FANCD2 identified by bioinformatics analysis. Further analysis of only hsa-miR-29c was incorporated in our following study, as this miRNA deficiency was closely associated with a more favorable prognosis and reversely correlated with FANCD2 mRNA expression, implying that hsa-miR-29c is more likely to modulate FANCD2 mRNA stability. This hypothesis is supported by previous studies, reporting that miR-29c plays a tumor-suppressive role in various cancers, such as hepatocellular carcinoma (Wu et al., 2019), non-small-cell lung cancer (NSCLC) (Sun et al., 2018), endometrial cancer (EC) (Li et al., 2019), colon cancer (Chen et al., 2017), prostate cancer (Li et al., 2018), and pancreatic cancer (Lu et al., 2021). For instance, overexpression of miR-29c-3p in HCC can activate large tumor suppressor gene 1 (LATS1) expression and demethylation of LATS1 *via* DNMT3B, thereby impeding HCC cell tumor growth and migration both *in vitro* and *in vivo* (Wu et al., 2019). Intriguingly, this is in agreement with other results reported by SW Nam’s group (Bae et al., 2014). These data suggest that FANCD2 is possibly a direct target of miR-29c-3p in HCC.

To date, extensive evidence has shown that lncRNAs could act as a molecular sponge for miRNAs in cancers *via* the ceRNA mechanism to modulate ferroptosis. For example, lncRNAs HULC in HCC induced ATF4 stability by competitively binding to miR-3200-5p, resulting in the inhibition of ferroptosis (Guan et al., 2022). Among these lncRNAs, we found that nine possible lncRNAs in HCC were screened through the starBase database, and the high activity of both DUXAP8 and LINC00511 within HCC had poorer survival outcomes. Interestingly, we observed that both lncRNAs displayed a positive correlation with FANCD2 expression but were opposite to miR-29c-3p. Our result further supports previous observations that DUXAP8 (Liu et al., 2021) and LINC00511 (Hu et al., 2020) act as ceRNA for miR584-5p together with miR195, further contributing to HCC cell proliferation, invasion, and migration, and DUXAP8 has also been reported to be ferroptosis-associated lncRNA (Xing et al., 2021). Overall, these data uncover that this lncRNA functions as the molecular sponge in HCC impinged on ceRNA machinery.

Tumor-infiltrating immune cells have generally emerged as critical controllers of the immunological response, as they are surrounded by various infiltrating inflammatory cells that can be exploited to modulate ferroptosis-inducing resistance in tumor cells or mediate antitumor immunity (Whiteside, 2002; Wattenberg and Beatty, 2020; Zhao et al., 2020). For example, CD4^+^ T cells represent a major component of the adaptive immune system, establishing effective antitumor properties (Togashi et al., 2019; Oh and Fong, 2021). Indeed, we also found that FANCD2 in HCC was strongly associated with the high infiltration of CD4^+^ T cells. Apart from this, it harbors the same trend in the infiltration of neutrophils, purity, macrophages, CD8^+^ T cells, dendritic cells, and B cells. These data demonstrate that FANCD2 is an essential component of the adaptive immune system against HCC cells. Immunotherapy has been shown to be the backbone of effective treatment against cancer cells. Its blockade of the interaction between ICIs, such as CTLA-4, PD-1, and PD-L1 antibodies, and their ligands with IC inhibitors has been approved to treat several types of tumors, depending on the results of the trials (Sharma and Allison, 2020). Importantly, FANCD2 in HCC revealed a similar trend to the aforementioned three checkpoints, which has been successfully validated by IPS analysis, indicating that it might exert a pivotal role in restoring immune cytotoxic activity.

Although GSEA was explored to decipher the tumor-associated and even tumor-promoting roles of FANCD2, encompassing the cell cycle, p53 signaling pathway, mTOR signaling pathway, DNA replication, and TGF-beta signaling pathway, there are some restrictions of the current study that need to be highlighted. First, gathering more robust data or samples of HCC is needed to verify the tumor-promoting role of FANCD2. Furthermore, despite using comprehensive and deep *in silico* analysis, we provided few experiments with meaningful insights into the DUXAP8-miR-29c-FANCD2 and LINC00511-miR-29c-FANCD2 axes *via* the ceRNA network and the effect of FANCD2 on antitumor immunity.

In summary, this is the first study to report that ncRNA-regulated FANCD2 in HCC plays a tumor-promoting role based on the starBase database, HCCDB, TIMER database, and GEPIA database, indicating that it can be employed as a potential prognostic marker. In addition, our studies reveal that this ferroptosis-associated regulator harbors a strong association with HCC immunity, including immune infiltration cells, immune infiltration markers, and immune checkpoints, suggesting its significant immunological antitumor value. Thus, FANCD2-mediated ceRNA offers a promising strategy for novel targeted HCC therapies.

## Data Availability

Publicly available datasets were analyzed in this study. These data can be found at: (https://xena.ucsc.edu/) and (http://starbase.sysu.edu.cn/).

## Supplementary material

Supplementary Figure S1 Prediction of DUXAP8 and LINC00511 subcellular localization using iLoc-lncRNA.

Supplementary Figure S2 Comprehensive analysis of FANCD2 in HCC. **(A)** Differential expression of FANCD2 across cancers according to the HCCDB. **(B)** Differential expression of FANCD2 in multiple HCC cell lines according to the CCLE database. **(C,D)** Kaplan‒Meier survival analysis of FANCD2 in HCC through the HCCDB.
