# Supplementary figures and images for: Integrative analyses of prognosis, tumor immunity, and ceRNA network of the ferroptosis-associated gene FANCD2 in hepatocellular carcinoma
Source: Front Genet. 2022 Sep 29;13:955225. doi: 10.3389/fgene.2022.955225 (PMC9557971; doi:10.3389/fgene.2022.955225)

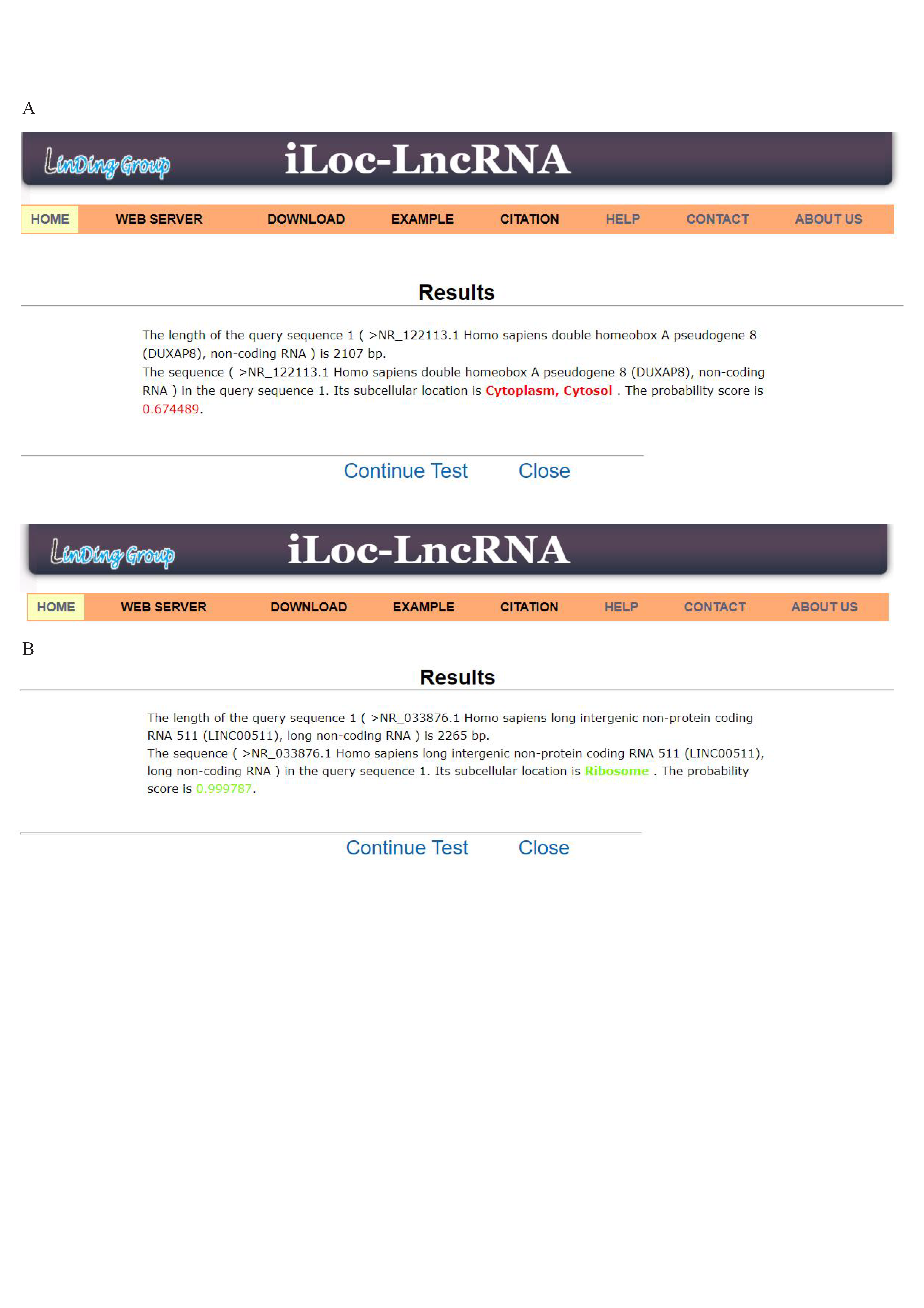

Supplement: Supplementary file 1 [file Image2.tif]

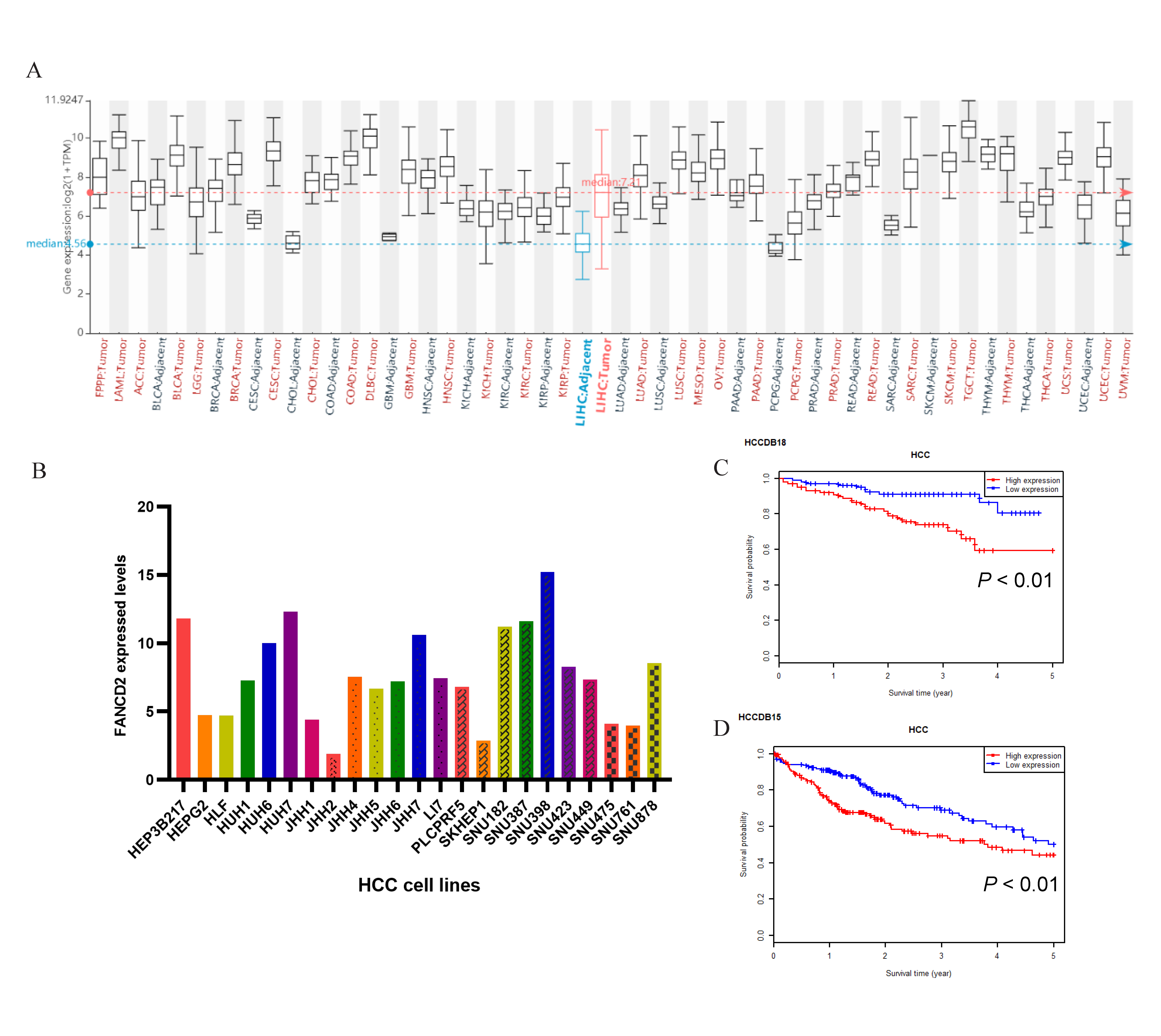

Supplement: Supplementary file 2 [file Image1.tif]
